# First report of bloodstream infection caused by *Apiotrichum veenhuisii* in a patient with acute lymphoblastic leukemia

**DOI:** 10.3389/fcimb.2025.1687957

**Published:** 2025-11-11

**Authors:** Yifeng Liu, Meng Li

**Affiliations:** 1 Department of Clinical Laboratory, The First Affiliated Hospital of Guangxi Medical University, Nanning, China; 2 Key Laboratory of Clinical Laboratory Medicine of Guangxi Medical University, Education Department of Guangxi Zhuang Autonomous Region, Nanning, China

**Keywords:** *Apiotrichum veenhuisii*, bloodstream infection, acute lymphoblastic leukemia, morphology, antifungal therapy, whole genome sequencing

## Abstract

*Apiotrichum veenhuisii* (*A. veenhuisii*), a type of yeast found widely in nature, is a rare pathogen that induces human infection globally. Here, we report a case of a female patient with recurrent B-cell acute lymphoblastic leukemia (B-ALL) who developed severe febrile neutropenia following systemic chemotherapy. In the meantime, a certain species of fungus was repeatedly detected in her peripheral blood and bone marrow cultures. This strain was ultimately identified as *A. veenhuisii* through morphological examination and molecular genetic analysis. Fungemia was therefore diagnosed. After amphotericin B and voriconazole treatments, the patient’s symptoms were resolved remarkably. No recurrent B-ALL was found in her bone marrow at 3 months of follow-up. This is the first evidence worldwide of bloodstream infection caused by *A. veenhuisii* in humans as far as we know. A precise aetiological diagnosis guides us on the correct path of antifungal treatment at a very early stage, making the patient recover with a favorable prognosis. Further functional annotation and phylogenetic analysis were performed after the whole genome was sequenced. This may help us better understand the biological characteristics and evolutionary relationships of this species.

## 1 Introduction

Fungaemia, a severe healthcare-associated opportunistic infection characterized by the presence of fungi in the bloodstream, has emerged as a significant clinical concern due to its rising incidence and high associated mortality (Flores-Delgado et al., 2024). Previously, *Candida* spp. was the most prevalent causative agent, accounting for over 80% of cases in China (Xiao et al., 2025). However, recent surveillance has revealed a concern that the detection rate of rare fungal pathogens, such as *Sporopachydermia lactativora* and *Trichosporon inkin*, is gradually rising in recent years (Zheng et al 2024; Wu, 2025).

*Apiotrichum veenhuisii* (*A*. *veenhuisii*), formerly known as *Trichosporon veenhuisii*, is a fungus whose classification has changed several times. It is currently reclassified in the genus *Apiotrichum* based on an extensive taxonomic study carried out in 2015 (Liu et al., 2015). *A*. *veenhuisii* was first isolated from buffalo dung by Italian scientists in 1985 (Middelhoven et al., 1985). It is often considered to be hypovirulent, which is attributed to its low biofilm formation and minimal secretion of proteinase, phospholipase, and hemolysin (Lara et al., 2019). Meanwhile, it also utilizes various carbon and nitrogen sources for growth, exhibiting metabolic diversity (Middelhoven et al., 2000).

Whole genome sequencing (WGS) is a transformative technology that enables the comprehensive determination of an organism’s complete DNA sequence. In microbiology, WGS serves as a cornerstone for elucidating genetic diversity, evolutionary mechanisms, and functional potential (Mustafa, 2024). However, the whole genomic sequence of *A*. *veenhuisii* clinical strain has not been updated since the original assembly of type strain JCM 10691 (GenBank ID: GCA_001600595) released by Japanese scientists in 2016.

In this study, we describe the first case worldwide of bloodstream infection (BSI) caused by *A*. *veenhuisii* in a patient with B-cell acute lymphoblastic leukemia (B-ALL). Besides the antifungal susceptibility patterns and typical morphological figures, we also incorporate virulence factors, drug resistance genes, and phylogenetic analysis based on the WGS. These data not only aid in accurate identification and guide clinical treatment, but also make beneficial attempts to seek a reference point for further epidemiological surveillance of *A*. *veenhuisii* infections in humans.

## 2 Case report

### 2.1 Clinical course

A 26-year-old female patient, who resided in Nanning City and worked as a technical staff in an Internet company, with a 7-year history of B-ALL presented to the hematology clinic owing to general weakness for a month. No fever, cough, sleep disorder, or loss of weight was noticed in the meantime. Blood test revealed the low levels of hemoglobin (62.00 g/L), prealbumin (160.30 mg/L), white blood cell count (2.71×10^9^/L), and percentage of neutrophil (12.80%), which suggested moderate anemia and malnutrition. Physical examination was notable for pale lips, tachycardia (heart rate 113 beats/min), and underweight (body mass index 16.6 kg/m^2^). Results of bone marrow cytology inspection showed that the percentages of lymphoblasts (36.50%) and prolymphocytes (58.00%) increased significantly, which demonstrated ALL recurrence. After excluding all contraindications, a 15-day of systemic chemotherapy was administered with the combination of vinorelbine (V), daunorubicin (D), cyclophosphamide (C), prednisolone (P), and L-asparaginase (L), namely the VDCPL protocol (**Supplementary Figure 1**).

### 2.2 Microbiological findings

**Figure 1:**
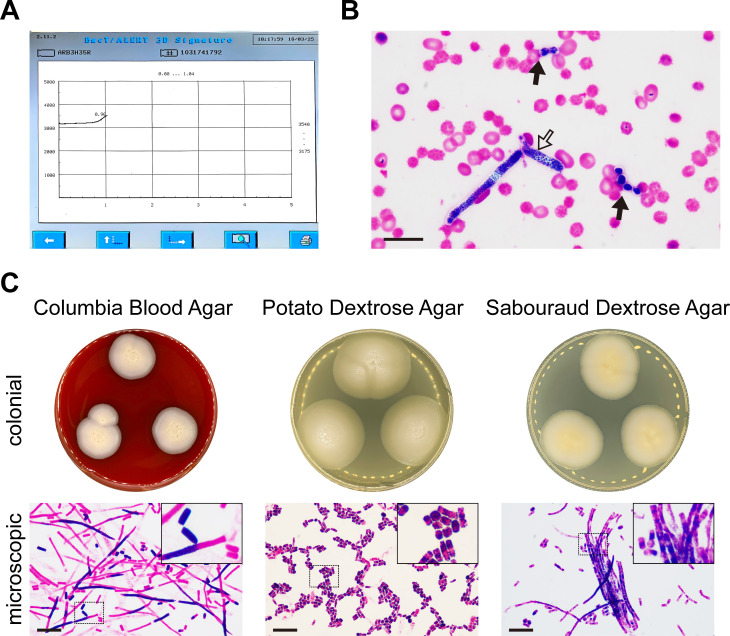
*Apiotrichum veenhuisii* in positive blood culture. **(A)** The growth curve of the microorganisms was plotted by the automated blood culture system that recorded the reflectance units of the culture mediums in real time over a 5-day period. **(B)** Representative image of the blastospores (solid arrows) and arthroconidia (hollow arrow) in smear from positive blood culture was captured under optical microscope after Gram staining. Bar, 20 μm. **(C)** The colonial and microscopic morphology of *Apiotrichum veenhuisii* grew in different culture mediums. The hyphae and spores were observed under optical microscope after Gram staining. The upper right corners show the partial magnification of sections marked with dashed boxes. Bar, 20 μm.

During the therapy, the patient experienced an episode of mild fever. Pathogens, such as *Escherichia coli* (*E. coli*), *Klebsiella pneumoniae* (*K. pneumoniae*), and *Stenotrophomonas maltophilia* (*S. maltophilia*), were detected in her blood. Fortunately, the prompt antimicrobial administration ensured the rapid eradication of these above bacteria. However, the patient developed severe febrile neutropenia 4 days after the end of treatment. The pattern was typical of remittent fever (**Supplementary Figure 1**). The growth curve of blood culture was monitored with an increasing trend after 24 hours of incubation (**Figure 1A**). Both the blastospores (**Figure 1B**, solid arrows) and arthroconidia (**Figure 1B**, hollow arrow) were observed in the smear from the positive blood culture. The colonial and microscopic morphology varied from different culture mediums (**Figure 1C**). On the Columbia blood agar (CBA), the colonies appeared flat, dry, and rough-edged, with slight hyphae growth. Although the length of hyphae varied, their thickness was basically the same under microscopic observation. On potato dextrose agar (PDA), the colonies were bulging and moist, with smooth and neat edges that shaped like a bun. Microscopically, the arthroconidia were fragmented and diffused. They were stumpy and usually exhibited opposite Gram staining properties at their two poles. Typical morphology of colonies on Sabouraud dextrose agar was an omelette-like appearance with a yellow center surrounded by white. Their humidity was between those on CBA and PDA. Both hyphae and arthroconidia were found under the microscope, which were clustered together like bamboos or bundles (**Figure 1C**).

In terms of other laboratory findings, the patient also exhibited persistent hyperthermia, pancytopenia, decreased counts of natural killer cells, and increased concentrations of serum ferritin and soluble CD25. These five points all met the diagnostic criteria of hemophagocytic lymphohistiocytosis (HLH) issued in 2004 (Henter et al., 2007). According to the latest guideline, fulfilling 5 out of 8 criteria is sufficient for HLH diagnosis (Henter et al., 2024). We suspected that the patient’s HLH was probably triggered by this unknown fungal infection.

To identify this causative organism, we first tried to use matrix-assisted laser desorption ionization-time of flight mass spectrometry (MALDI-TOF MS). To our surprise, both MALDI-TOF MS produced by bioMérieux (Marcy l’Etoile, France) and Zybio (Chongqing, China) companies were able to characterize this fungus with two similar patterns of spectral profiles. The characteristic peaks were detected at around 3550 and 7100 daltons for one type (upper panels of **Supplementary Figures 2A, B**, green arrows), and at around 6150 and 9420 daltons for the other (lower panels of **Supplementary Figures 2A, B**, blue arrows). But unfortunately, none of them gave credible identification results.

We next turned to internal transcribed spacer (ITS) sequencing. The strain was ultimately identified as *A*. *veenhuisii* with 99.19% and 99.80% of identity in the NCBI rRNA/ITS and MYCOBANK Fungal databases, respectively. A diagnosis of fungemia was thus made. The sequences were later deposited in GenBank under the accession number PV945980.

The minimum inhibitory concentrations (MICs) of antifungal agents against *A*. *veenhuisii* were determined by antimicrobial susceptibility testing (AST) *in vitro*. We only reported the MIC values without interpretation, since neither clinical breakpoint nor epidemiological cut-off value was available for *Apiotrichum* spp until now. As shown, the lowest MICs were found in amphotericin B (0.5 μg/mL) and voriconazole (0.06 μg/mL), while echinocandins, such as micafungin and caspofungin, remained at high levels (**Supplementary Table 1**).

### 2.3 Treatment and outcome

**Figure 2:**
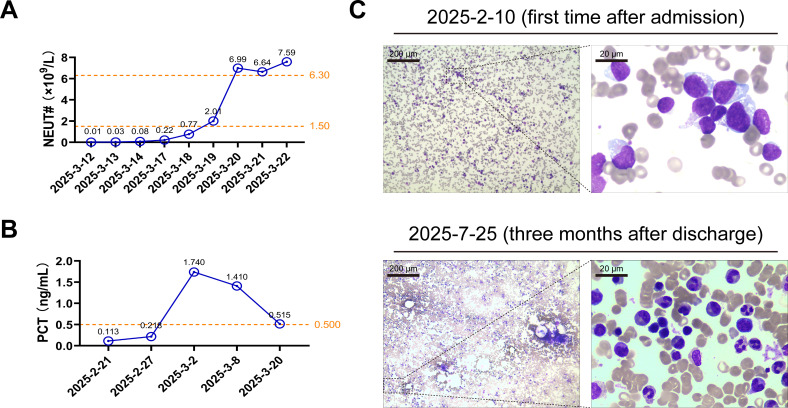
The patient received an optimistic outcome after treatments. **(A, B)** The patient’s peripheral blood neutrophil counts (NEUT#) and serum procalcitonin (PCT) concentrations were measured on the indicated dates. The exact values are marked above each point. The orange dotted lines represent the edges of reference intervals. **(C)** The bone marrow smears obtained from the patient on the indicated date were examined under the optical microscope following Wright-Giemsa staining. Representative images of the partial magnification below show the blood cells in detail.

Based on the results of AST, the patient was timely prescribed intravenous liposomal amphotericin B (150 mg, once daily) and voriconazole (200 mg, twice daily) for anti-infection. The dexamethasone (15 mg, once daily) for HLH treatment. After 2 weeks of efforts, the patient’s temperature (**Supplementary Figure 1**), procalcitonin (**Figure 2A**), and neutrophil count (**Figure 2B**) returned to normal. Finally, she was discharged after the infection had been successfully controlled. Her bone marrow was assessed as being in complete remission after 3 months of follow-up, based on active granulocyte proliferation and a significant decrease in the percentage of immature lymphocytes compared to the levels observed in the first examination following admission (**Figure 2C**).

## 3 WGS-based gene analysis

Upon completion of the WGS, the DNA sequences were deposited in the GenBank under the accession number JBPYUH000000000 (BioProject ID: PRJNA1292434).

To estimate the possible inactivation effects of *A*. *veenhuisi* on antibacterial agents, we first aligned the protein sequences using the NCBI Blast+ software (v2.2.28) with E-value cut-off of 10^-5^ against the comprehensive antibiotic database (CARD) and mycology antifungal resistance database (MARDy). As shown in **Supplementary Table 2**, a total of 17 antibiotic resistance genes were identified, which were distributed among 11 primary ontologies. The greatest number of ontologies occurred in cephalosporin, penam, and peptide antibiotic resistance, with 6 primary ontologies attributing to the abnormal expression of antibiotic efflux. Glycopeptide antimicrobial resistance due to antibiotic target alteration came a close second, supported by 4 primary ontologies. However, there was no genetic event related to antifungal resistance detected, which is consistent with the results of AST in **Supplementary Table 1**.

Next, the Database of Fungal Virulence Factors (DFVF) was accessed to gather genes linked to potential virulence factors in fungi. Results were retained only for hits with the item “Identity” above 80% as well as “Disease-Host” included animal or vertebrata. As shown in **Supplementary Table 3**, 15 kinds of virulence factors were filtered, most of which were structural proteins (such as HIS3 and TUB1), kinases (such as MPK1and RAS1), and metabolic enzymes (such as UGD1, FKS1, UXS1, and GNO1). These factors participated in cellular viability, signal transduction, and amino acid/carbohydrate metabolic processes, and exhibited significant associations with cryptococcosis and invasive candidal disease.

To further investigate the genetic relationship and phylogenesis of *A*. *veenhuisi* at higher resolution, the ITS sequences of strains were obtained from gene prediction using WGS and subsequently aligned with ITS sequences of other strains collected in NCBI Nucleotide Database with an identity threshold set at over 95%. Evolutionary history was inferred using the maximum likelihood method in MEGA 7 software (v7.0.26). The phylogenetic tree was drawn to scale and then beautified using the online tool TVBOT (v2.6.1) at https://www.chiplot.online. As shown in the phylogenetic tree, most enrolled strains were *Apiotrichum* spp. *A*. *veenhuisi* clinical strain (GenBank ID: PV945980, ITS region) had a longer branch length than its type strain JCM 10691 (GenBank ID: AF414693, ITS region) (0.00513 vs 0.00255). They formed a separate and well-supported cluster (97% bootstrap). These results implied that for *A*. *veenhuisi*, the clinical strain has undergone more significant evolutionary changes since diverging from the common ancestor compared to the type strain.

## 4 Discussion

This case represents the first confirmed instance of *A. veenhuisii* fungemia in the world. The yeast *A. veenhuisii*, noted for its arthroconidia and morphological diversity, is an environmental organism but poses risks to immunocompromised individuals (Ilyas and Sharma, 2017; Spallone and Schwartz, 2021).

Human infection caused by *A*. *veenhuisii* is extremely rare globally. The only reported case is a Brazilian teenager who relapsed with acute myeloid leukemia after receiving bone marrow transplantation during adjuvant chemotherapy. The strain was isolated from disseminated skin lesions, while repeated blood cultures remained negative. The patient ultimately died from refractory septic shock (Lara et al., 2019).

In this report, we reviewed the patient’s culture records with positive results during her current hospital stay. We discovered that, since the first positive culture isolated from the patient’s peripheral blood on March 4^th^, *A*. *veenhuisii* was repeatedly detected in her peripheral blood and bone marrow several times (**Supplementary Figure 1**). This implied that, first, the detection of *A*. *veenhuisii* was not a false positive caused by environmental or operational contamination. Second, the patient’s condition grew worse. This fungus is hard to eradicate once it infects a patient with immunodeficiency.

Admittedly, the first case worldwide of human BSI caused by *A*. *veenhuisii* we documented here is more aggressive and complicated than the case in Brazil. However, the patient was ultimately rescued from a life-threatening situation. This success cannot be achieved without the integration of multiple methodologies, such as traditional microbiological morphology, an automated blood culture system, and advanced molecular biotechnology. Furthermore, timely communication and active cooperation among multidisciplinary team members, including clinicians, pharmacists, and microbiology specialists, also play a critical role in medication decisions.

Fungi and bacteria frequently coexist and interact across diverse environments, such as soil and the human gut. These interactions, driven by direct contact or chemical signals, significantly influence the microorganisms’ growth, behavior, and pathogenicity, playing a crucial role in ecosystem functions and host-associated communities (Pawlowska, 2024). In this study, we annotated the antibiotic resistance genes of *A*. *veenhuisii* clinical strain by WGS. The results showed that overexpression of genes encoding efflux pump that might be responsible for cephalosporin, penam, and peptide antibiotic resistance were detected in the genome of *A*. *veenhuisii*, which reminds us to use these kinds of antibiotics with caution when patients are suspected of possible *A*. *veenhuisii* infection. *A*. *veenhuisii* is quite likely to increase the resistance of the surrounding bacteria to these antibiotics, thus rendering antibiotic therapy ineffective (**Supplementary Table 2**).

Additionally, as shown in **Supplementary Table 3**, genes encoding structural proteins, kinases, and metabolic enzymes were enrolled in the annotation analysis of potential virulence factors. From this, we hypothesize that signalling pathways that regulate cell division and metabolism may also be involved in virulence regulation. Their abnormal activation possibly triggers the strain to become hypervirulent. Besides, we also noticed that FKS1 was matched in DFVF (**Supplementary Table 3**). Although the identity rate was only 84.2%, it precisely implied the possibility of a mutation in this gene. FKS1 is an enzyme which is crucial for the synthesis of 1, 3-β-glucan, a major polysaccharide that provides structural integrity to the fungal cell wall (Hu et al., 2023). Its mutation was most commonly found in the *Candida* species, leading to echinocandins resistance by disturbing the synthesis of fungal cell walls (Tian et al., 2024), which might also account for the high MIC values of echinocandins against *A*. *veenhuisii* in our study. Future research will focus on identifying the exact mutation sites to obtain more reliable evidence. It is reported that both the cell wall of *Trichosporon* spp. and the capsule of *Cryptococcus* spp. contain secretory glucuronoxylomannan with similar structure, which can cause false positive test results and even lead to fatal outcomes owing to the cross-reactivity (Goncalves et al., 2021; Lin et al., 2025). *Trichosporon* spp. and *Apiotrichum* spp. are members of basidiomycetous yeasts and are close relatives in evolution. These conclusions may provide a rational explanation for why most of virulence factors detected in our work were related to cryptococcosis (**Supplementary Table 3**).

**Figure 3:**
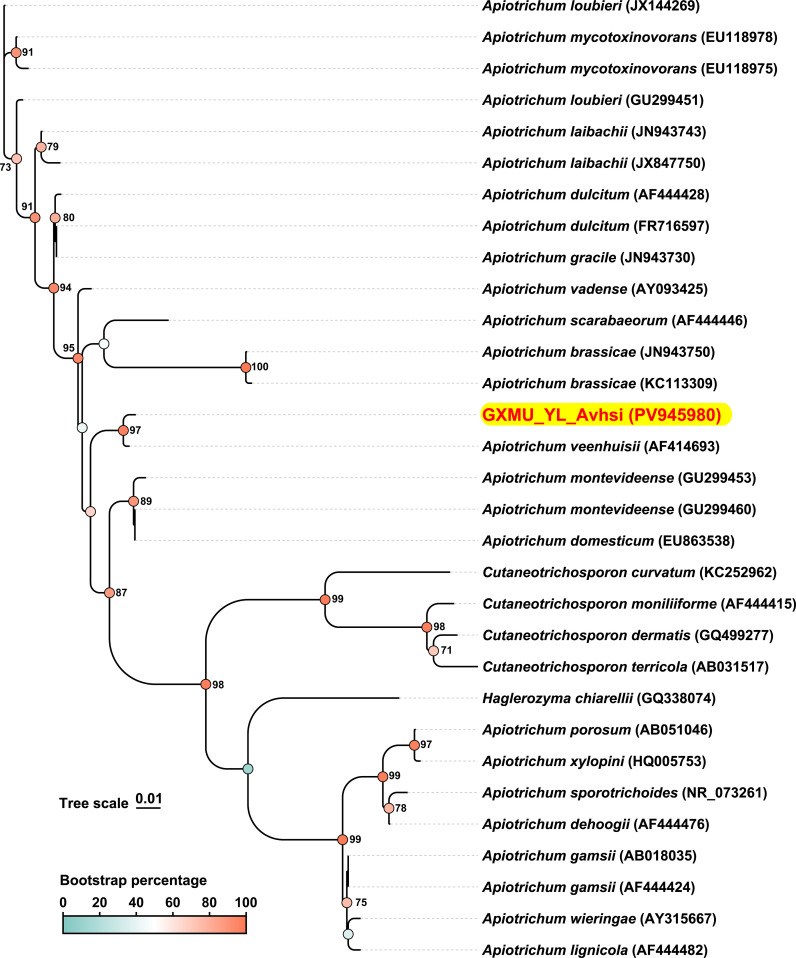
Internal transcribed spacer (ITS)-based phylogenetic tree generated by whole-genome sequencing. The ITS sequence of *Apiotrichum veenhuisii* obtained in this study is presented with red-on-yellow highlighting. Accession numbers of sequences in GenBank are given in brackets behind the strain names. The color represents the bootstrap percentage which was based on 1000 replicates. Only the values greater than or equal to 70% are labeled as percentages at branching points. Scale bar indicates 0.01 substitutes per nucleotide position.

ITS region, a segment of non-coding RNA, lies between the 5.8S, 18S, and 28S rRNA in fungi. It is regarded as the formal fungal DNA barcode and is widely adopted as a standard marker for species-level identification, phylogenetic inference, and diversity assessments across diverse eukaryotic lineages (Tekpinar and Kalmer, 2019). In this study, we investigated the genetic relationship and phylogenesis of *A*. *veenhuisi* at a higher resolution by WGS. As shown in **Figure 3**, PV945908 (*A*. *veenhuisi* clinical strain) was further evolved than AF414693 (*A. veenhuisi* type strain) from the common node they shared. This result reminds us that we should remain highly vigilant about changes in virulence and drug resistance that may occur during strain evolution.

To summarize, we detailed distinctive morphological characteristics and introduced an effective antifungal therapy against *A*. *veenhuisii* in BSI. More importantly, we also include the gene functional annotation and evolutionary relationships based on WGS. We aim to share these insights with the global medical community to enhance their capacity for successful management of similar cases, and meanwhile, lay the experimental foundation for in-depth research of *A*. *veenhuisii* in future clinical settings.

## Data Availability

The datasets presented in this study can be found in online repositories. The names of the repository/repositories and accession number(s) can be found below: https://www.ncbi.nlm.nih.gov/genbank/, PV945980 https://www.ncbi.nlm.nih.gov/genbank/, JBPYUH000000000.
