# Supplementary material for: First report of bloodstream infection caused by Apiotrichum veenhuisii in a patient with acute lymphoblastic leukemia
Source: Front Cell Infect Microbiol. 2025 Nov 11;15:1687957. doi: 10.3389/fcimb.2025.1687957 (PMC12644028; doi:10.3389/fcimb.2025.1687957)
Supplement: Supplementary file 1 [file DataSheet1.pdf]

Supplementary Material

First Report of Bloodstream Infection Caused by Apiotrichum veenhuisii in a Patient with Acute Lymphoblastic Leukemia

Yifeng Liu[1,2], Meng Li[1,2*]

[1] Department of Clinical Laboratory, The First Affiliated Hospital of Guangxi Medical University, Nanning, China

[2] Key Laboratory of Clinical Laboratory Medicine of Guangxi Medical University, Education Department of Guangxi Zhuang Autonomous Region, Nanning, China

*** Correspondence:**
Meng Li
gxmulimeng@foxmail.com

Supplementary Table 1. Antifungal susceptibility profile of *Apiotrichum veenhuisii*.

Antifungal agent	Minimum inhibitory concentration (μg/mL)
5-Fluorocytosine	1
Amphotericin B	0.5
Voriconazole	0.06
Itraconazole	0.5
Fluconazole	1
Micafungin	>8
Caspofungin	>8

Footnote: Minimum inhibitory concentrations (MICs) were determined by broth microdilution method. According to the M27 guideline (3rd Edition) issued by Clinical and Laboratory Standards Institute, no breakpoint is available for *Apiotrichum* spp until now. Therefore, we only reported the MIC values but not the interpretation results in this assay.

Supplementary Table 2. Distribution of putative resistance genes in *Apiotrichum veenhuisii*.

Accession	Drug class	Resistance mechanism	Gene number
ARO:3003942	cephalosporin, penam, peptide antibiotic	antibiotic efflux	6
ARO:3002947	glycopeptide antibiotic	antibiotic target alteration	2
ARO:3002522	aminocoumarin antibiotic	antibiotic efflux	1
ARO:3002892	tetracycline antibiotic	antibiotic efflux	1
ARO:3002943	glycopeptide antibiotic	antibiotic target alteration	1
ARO:3002944	glycopeptide antibiotic	antibiotic target alteration	1
ARO:3003730	mupirocin-like antibiotic	antibiotic target alteration	1
ARO:3003950	nitroimidazole antibiotic	antibiotic efflux	1
ARO:3004036	tetracycline antibiotic	antibiotic efflux	1
ARO:3005345	diaminopyrimidine antibiotic	antibiotic target replacement	1
ARO:3007188	glycopeptide antibiotic	antibiotic target alteration	1

Supplementary Table 3. Distribution of putative virulence factors in *Apiotrichum veenhuisii*.

UniProt ID	Gene	Protein	Diseases	Gene number
Q5ADS0	UBI4	Polyubiquitin	invasive candidal disease	2
D2JLR3	HIS3	Histone H3	mycotoxins	2
P0CM55	CNB1	Calcineurin subunit B	cryptococcosis	1
Q58Z64	VAD1	ATP-dependent RNA helicase VAD1	cryptococcosis	1
C4MEZ6	MPK1	Mitogen-activated protein kinase	pulmonary cryptococcosis, basal meningitis, and cerebral cryptococcomas	1
Q9UVQ4	RAS1	Ras-like protein	cryptococcosis	1
O42766	BMH1	14-3-3 protein homolog	invasive candidal disease	1
Q96VU5	UGD1	UDP-glucose 6-dehydrogenase	cryptococcosis	1
Q5RZ66	TRR1	Thioredoxin reductase	cryptococcosis	1
Q8J2S9	FKS1	1,3-beta-glucan synthase	cryptococcosis	1
Q96UM1	TUB1	Tubulin beta chain	infection	1
Q7LJU0	UXS1	UDP-glucuronic acid decarboxylase 1	cryptococcosis	1
Q9P4E9	GSP1	GTP-binding nuclear protein GSP1/Ran	invasive candidal disease	1
Q6XVN4	GNO1	S-(hydroxymethyl)glutathione dehydrogenase	cryptococcosis	1
Q9P8W9	CPA1	Peptidyl-prolyl cis-trans isomerase	cryptococcosis	1

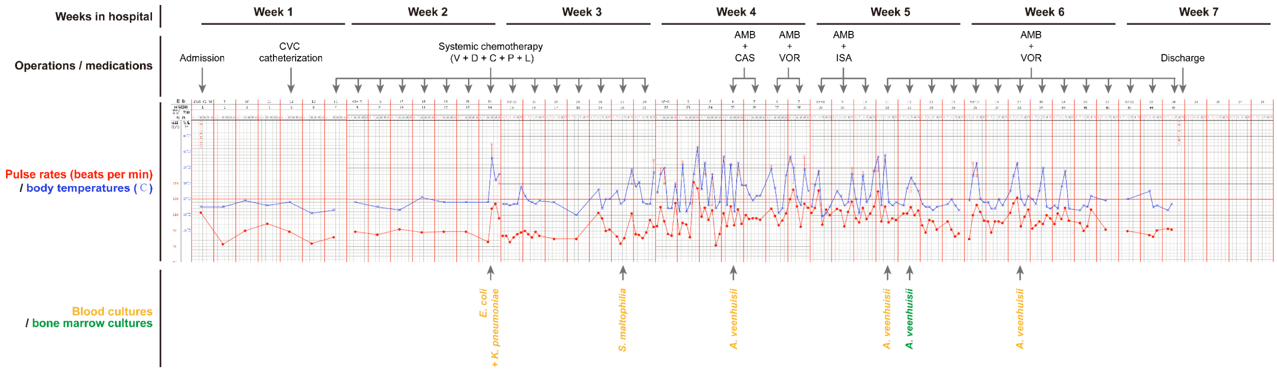

Supplementary Figure 1. Medical records of the patient during his hospitalization. The timeline documented the main medical operations and medications during the patient's hospitalization. Downward arrows indicate the execution dates. The daily pulse rates (beats/min) and body temperatures (℃) were recorded and plotted into line chart in red and blue colors, respectively. The pathogens isolated from blood and bone marrow are marked in yellow and green, respectively. Upward arrows indicate the dates of positive alarms issued by the automatic monitoring system. Abbreviations: CVC, central venous catheter; V, vinorelbine; D, daunorubicin; C, cyclophosphamide; P, prednisolone; L, L-asparaginase; AMB, amphotericin B; CAS, caspofungin; VOR, voriconazole; ISA, isavuconazole.

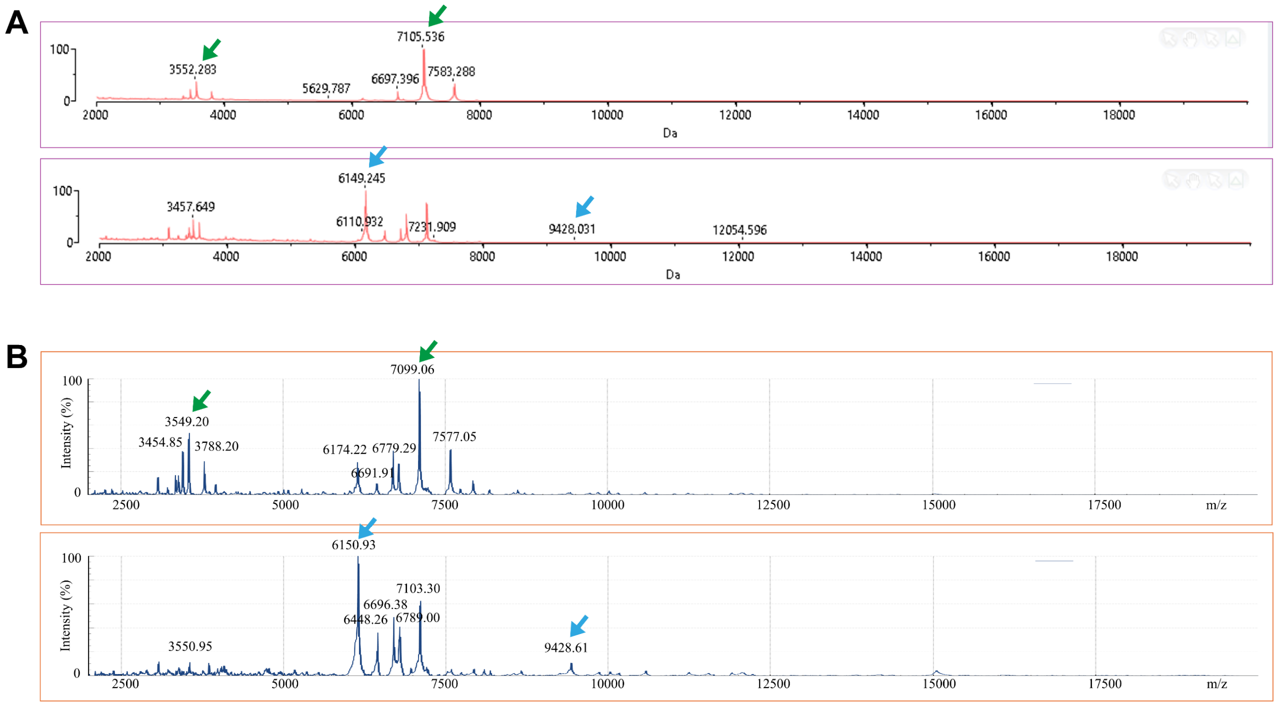

Supplementary Figure 2. The mass spectral profiles of *Apiotrichum veenhuisii* acquired by matrix-assisted laser desorption ionization-time of flight mass spectrometry (MALDI-TOF MS). Two types of spectral profiles of *Apiotrichum veenhuisii* with similar characteristic peaks were acquired by MALDI-TOF MS produced by (A) bioMérieux and (B) Zybio company. The potential characteristic peaks in the first and second types of spectral profile are indicated by the green and blue arrows, respectively. The horizontal and vertical coordinate represents mass-to-charge ratio (m/z) and relative intensity of ion current (%), respectively.
